# The Effect of Gluten-Free Diet on Health and the Gut Microbiota Cannot Be Extrapolated from One Population to Others

**DOI:** 10.3390/nu10101421

**Published:** 2018-10-04

**Authors:** Jose F. Garcia-Mazcorro, Giuliana Noratto, Jose M. Remes-Troche

**Affiliations:** 1 Instituto de Investigaciones Medico Biológicas, Universidad Veracruzana, Calle Agustín de Iturbide, Salvador Díaz Mirón, Veracruz 91700, Mexico; josegarcia_mex@hotmail.com; 2 Department of Nutrition and Food Science, Texas A&M University, 2253 TAMU, College Station, TX 77843, USA; gnoratto@tamu.edu

**Keywords:** celiac disease, gluten-free diet, gut microbiota

## Abstract

Gluten-related disorders (GRD) affect millions of people worldwide and have been related to the composition and metabolism of the gut microbiota. These disorders present differently in each patient and the only treatment available is a strict life-long gluten-free diet (GFD). Several studies have investigated the effect of a GFD on the gut microbiota of patients afflicted with GRD as well as healthy people. The purpose of this review is to persuade the biomedical community to think that, while useful, the results from the effect of GFD on health and the gut microbiota cannot be extrapolated from one population to others. This argument is primarily based on the highly individualized pattern of gut microbial composition and metabolic activity in each person, the variability of the gut microbiota over time and the plethora of factors associated with this variation. In addition, there is wide variation in the composition, economic viability, and possible deleterious effects to health among different GFD, both within and among countries. Overall, this paper encourages the conception of more collaborative efforts to study local populations in an effort to reach biologically and medically useful conclusions that truly contribute to improve health in patients afflicted with GRD.

## 1. Introduction

Human beings are superorganisms or holobionts (i.e., hosts with associated life forms) that have evolved over millions of years collectively as a unit, yet independently [1]. From all microbial niches in the body, the digestive tract has received the mostattention in part due to its role in health and immunity [2]. Many different host-associated (e.g., age, sex, health status) and environmental factors are known to affect the composition of the gut microbiota but growing evidence suggests that diet is one of the main contributors [3,4,5]. Diet is particularly relevant in newborns and infants, where nutrition is not only vital for growth and development but can also have life-long consequences, a phenomenon closely linked to the gut microbiota [6,7]. 

The gut microbiota has been studied in a context of health and disease for over a century now. Generally, the gut microbiota is in balance with its host and shows certain resilience to change from one state to another (e.g., from healthy to diseased), although this phenomenon is still not well understood [8,9]. For example, while different diseases have been related to different states of “dysbiosis” of the gut microbiota (for example, allergies, inflammatory bowel diseases, diabetes, obesity and gluten-related disorders, see [10]), a cause–and–effect relationship can hardly be established in part because of the well-known high inter-individual variability, a phenomenon occurring even among closely related individuals [11]. Nonetheless, great progress has been achieved in understanding causal relationship between the host and its microbes [12]. 

The purpose of this review is to warn against extrapolation of results in the context of an effect of gluten-free diet (GFD) on health and the gut microbiota. Indeed, any metabolic response needs to be investigated specifically within population groups to increase our understanding of whether dietary treatments are effective in that group. Although the issue of extrapolation is true in all research in Nutritional Sciences, it is often overlooked and not emphasized enough among clinicians and clinical scientists.

## 2. Gluten-Related Disorders and Celiac Disease

Gluten-related disorders (GRD) comprise a variety of different disorders such as celiac disease (CD), non-celiac gluten sensitivity (NCGS), gluten allergy and others, where the body reacts negatively upon exposure to dietary gliadins, a class of proteins that are a component of gluten in wheat and other cereals. Clinically, GRD often range from mild presentations (such as asymptomatic CD) to very serious and life-threatening conditions in some people such as refractory CD and lymphoma [13]. 

From all GRD, CD has been the most studied and is currently considered to be the most common chronic enteropathy worldwide [14]. In people with CD, a significantly enhanced autoantibody response to the transglutaminase 2 (TG2) enzyme, also known as tissue transglutaminase (tTG), is a hallmark of the pathogenic process that primarily affects the architecture of the enterocyte lining of the small intestine [15] but can also affect other organs such as the liver, kidney, lymph nodes and muscles [16,17]. Interestingly, other autoantibodies may also be involved, especially in extraintestinal manifestations, such as anti-ganglioside, anti-synapsin I and anti-actin antibodies [18]. Patients with CD present either typical or atypical symptoms [19] and CD is believed to perpetuate other maladies and often presents simultaneously with other autoimmune diseases [20,21,22,23]. Despite the great progress in CD research, new key emerging findings suggest previously unknown features of CD pathogenesis, for example at the transcriptome level of immune cells [24].

The global prevalence of CD based on serologic test results is 1.4% and based on biopsy results is 0.7% [25]. The prevalence of CD varies with sex, age, and location and, in some regions and populations, it can be as high as 5.6% [26]. While different treatments are under study (e.g., using prolyl endopeptidases and vaccines [17,27]), the only effective treatment available to date for patients with CD and other GRD is a strict life-long gluten-free diet (GFD). Interestingly, GFD is being adopted worldwide by a growing number of people with and without GRD for weight control and the rather misconceived perception that this diet is healthier [28]. However, whether a GFD is healthier remains highly controversial (see Section 5.2).

## 3. GRD and the Gut Microbiota

The fact that genetic susceptibility is not determinant for the presentation of CD (30–40% of the population have the required genotype but the prevalence of CD is only about 1%) has prompted research to discover what other factors can predict the clinical manifestation of the disease [21]. For example, there is enough evidence to suggest that the gut microbiota (especially Bacteria) plays a role in the onset and clinical manifestations of CD [29,30,31,32,33,34,35,36,37,38] and probably other GRD. Although the exact mechanisms involved in the relationship of the gut microbiota and gut diseases are currently unknown (a relationship that is likely to be highly individualized as well), fellow colleagues have suggested an interesting proposal involving first a microbial dysbiosis (e.g., after antibiotic therapy), independent of gluten sensitivity, which then drives an activation of the innate immune system resulting in the secretion of pro-inflammatory molecules, epithelial barrier disruption, and an increased transfer of gluten peptides, a cascade that ultimately may lead to CD development [15]. Interestingly, CD may also be related with non-bacterial members of the gut microbiota such as yeasts [39,40], although the mechanisms may involve quite different mechanisms such as inter-kingdom interactions [41]. As in the case of other intestinal maladies, the main objective of these studies is to better understand the host–microbiota relationship during the disease (often compared to healthy counterparts), thus helping find new routes for treatment. For instance, the growing body of literature about host–microbiota in CD patients has prompted the use of some probiotics (e.g., *Lactobacillus* spp.) to treat GRD, particularly CD, with promising results (e.g., suppression of pro-inflammatory cytokines, reduction of mucosal damage, and enhanced production of SCFAs [31,42,43,44,45]). Other studies have shown a potential of probiotics to modulate the indigenous gut microbiota in patients with CD with inconsistent results [46,47].

## 4. Effect of GFD on the Gut Microbiome

**Figure 1:**
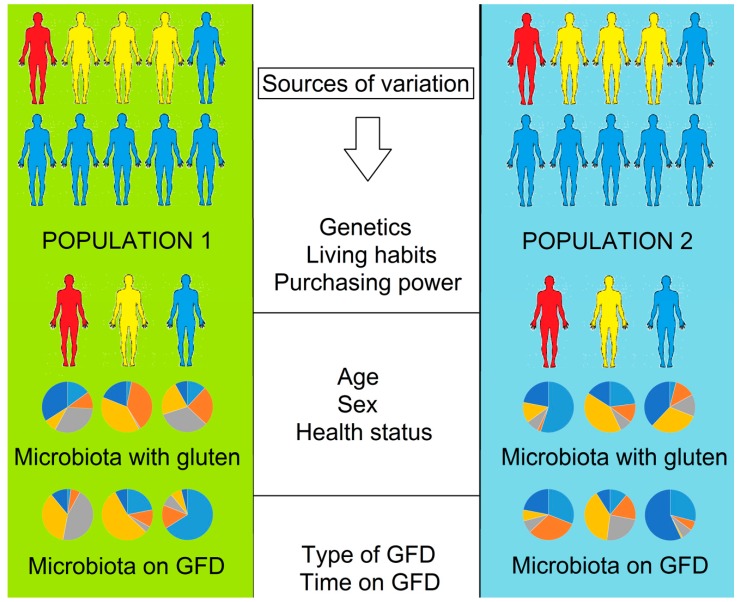
Results of the effect of gluten-free diet (GFD) on health and the gut microbiota cannot be extrapolated from one population to others. Genetic predisposition to CD is present in about 30–40% of the whole population (non-blue silhouettes) but affects clinically only about 1% of the population (red silhouettes). Each individual in either population harbors a highly specific microbiome in the gut (represented here by hypothetical data in pie charts where each color represents a different microbial group) that shows a unique pattern of change after consuming a GFD. The individualized microbiome and its response against dietary or therapeutic challenges is due to multiple sources of variation at the population (e.g., genetics andliving habits), individual (e.g., age andsex) and experimental (e.g., type and time on GFD) level.

The effect of GFD on the gut microbiome and related parameters has been studied in patients afflicted with GRD (particularly with CD) and in healthy subjects (Table 1). Please note that most of these studies have important limitations including small sample sizes and the use of low-throughput techniques (e.g., culturetechniques and non-sequencing based molecular techniques) that allow the analysis of a few bacterial groups that are not representative of the whole microbiota (Table 1, please note that great progress has been achieved in the field of gut microbiota culturomics [48]). The small sample sizes are particularly worrying because of the clinical variations of CD presentation. These limitations by themselves should be considered as warning signs by the biomedical community every time someone attempts to extrapolate results among different populations, especially in cases where there are patients involved because individuals are highly unique in terms of their gut microbiome (Figure 1).

Before discussing the issues with extrapolation and the main arguments against extrapolation of results, it is important to briefly discuss about the nature of microbes and their identification. First, while we tend to think that members of the same “species” should share a great deal of similar characteristics, this is far from being true for most (if not all) microbial species [62]. Second, when dealing with reference gene sequences (e.g., 16S rRNA gene sequences), it has been a long tradition to group these sequences into something we call Operational Taxonomic Units (OTUs), which are simply groups of sequences based on sequence similarity, based on the belief that a given OTU would comprise similar organisms. However, OTUs show extensive mixed phylogenetic and ecological signals [63] and in fact current trends suggest that OTUs should be replaced by exact sequence variants [64]. Third, there is extensive horizontal gene transfer (i.e., movement of genetic material between different organisms, for example genes associated with antibiotic resistance) among the many members of the gut microbiota that happens at mostly unknown rates [65], a phenomenon shaped principally by ecology rather than geography or phylogeny [66] that likely generates “new” microbes de novo. Fourth, there are different techniques to identify microorganisms (e.g., culture and culture-independent), each with its own advantages and disadvantages to truly depict the real microbial ecosystem inside the gut [67]. Finally, there are technical issues that are difficult to overcome, for example the fact that bacteria are not evenly distributed in stools [68], that mucus and lumen contain widely distinct microbial ecosystems [69], and that the microbiota is different throughout the intestinal tract and different fromwhat is found in feces [70]. 

## 5. The Issue of Extrapolation

The main objective of this paper is to critically argue against extrapolation in the context of an effect of GFD on health and the gut microbiota (Figure 1). Extrapolation of results from one population to another is incorrect and risky for various reasons, both strictly statistical and scientific. As it will become clear throughout this manuscript, this concern is particularly important in the context of gut microbial ecology, health and disease. On a recent review of the relationship between the gut microbiota and dietary nutrients, Shortt et al. [71] acknowledgedthe fact that animal-derived data can hardly be extrapolated to humans, and there is a well-known bias to choose male rodents in studies from different fields of science [72], including microbial ecology [3]. However, the problem with extrapolating results among human populations is barely mentioned in the literature, even in papers from our own research group [73]. We do not generally mention this because we consider it to be common knowledge and implicit in the results of our publications. However, we strongly believe that this concern should be discussed, especially within the context of health benefits derived from a change in the gut microbial ecosystem. 

### 5.1. Individuality and Over Time Variability of the Gut Microbiota

Each human being harbors a unique blend of trillions of microorganisms and viruses in the gut and other organs, and growing evidence suggest that colonization starts before birth [74]. The microbiota is not only highly different among individual subjects but it also shows a highly individualized response to environmental challenges such as antibiotic perturbation [75]. One study showed that variation in composition of the microbiota across different body sites was consistently larger than technical variability (e.g., PCR primers, 16S rRNA gene region, sequencing platform) across studies [76]. Overall, this means that the many different analyses showing inter-individual variation are biologically meaningful and not the result of technical artifacts. 

The question of why the microbiota is so unique in each individual deserves thorough examination. The first studies on the gut microbiota soon revealed that each subject harbors a unique blend of microbes [77]. Microbial carriage varies between subjects down to the species and strain level [78,79]. Virtually all host-associated and environmental factors can have an effect on an individual’s gut microbiota, either individually or collectively. Sex is also an important but often neglected topic in gut microbial ecology [80]. This is important because some GRD are known to be more prevalent in women [81]. On top of the well-known high inter-individual differences, there is considerable horizontal gene transfer happening inside the gut [64], which have the potential of increasing the uniqueness of each individual microbiome, and some microbes show bistable abundance distributions that are affected differently than the rest [82].

Another reason each individual is unique is because they live in vastly different geographic and sociocultural regions having unique foods and dietary habits. For example, Mexican people possess one of the most genome-wide variation, a fact that can affect biomedical traits as well as disease presentation, progression and response to treatment [83]. Interestingly, one seminal study about inflammatory bowel disease (IBD) and the gut microbiota showed that the patient’s geographical origin was strongly associated with disease presentation and involvement of specific microbes [84].

Different studies have evaluated the gut microbiota over short periods of time but very few studies have analyzed changes in the gut microbiota over long periods of time. One study showed a pronounced variability in an individual’s microbiota across months, weeks and even days, and that only a small fraction of all taxa appear to be present across all time points (in this study, 396 time points were analyzed [85]). Interestingly, baseline populations (i.e., before any major dietary or other change) can also predict the response of the gut microbiota in some situations [86]. 

### 5.2. Dietary Differences in Gluten-Containing and GFD 

Gluten-containing foods provide many nutrients (e.g., prebiotics such as inulin) which may not be equally abundant in gluten-free foods. The potential issues associated with GFD have been discussed since the 1950s [87] and the concern that a GFD could produce potentially adverse effects in the microbiota solely based on a marked reduction in intake of naturally occurring prebiotics has been raised [88]. On the one hand, a recent review showed that long-term morbidities associated with CD, such as nutritional deficiencies, impaired bone health, and reproductive abnormalities, can substantially improve after strict adherence to a GFD [89]. However, a study in Italy reveals an overall low nutritional quality of gluten-free bakery products [90], and gluten-free products contribute to imbalanced diets in children from Spain [91]. In addition, a recent review showed that reduced intake of calcium, vitamins and fiber as well as enhanced consumption of fat and carbohydrates have been consistently reported in patients on GFD [92]. Moreover, there is evidence that some gluten-free foods are not enriched and may be deficient in several nutrients, including dietary fiber, folate, iron, niacin, riboflavin, and thiamine [93,94], although this would not necessarily lead to dietary deficiency of these nutrients because other gluten-free foods such as vegetables, beef, eggs and cheese are rich in these compounds. Other studies evaluating the nutritional composition of processed gluten-free products have demonstrated higher levels of lipids, trans-fat, protein, and salt compared to their gluten-containing counterparts. Furthermore, recent evidence has shown that patients under a GFD are at risk of metabolic syndrome and hepatic steatosis [95] and the concerns regarding the association between micronutrient deficiencies and increased exposure to toxins such as arsenic in GFD [96]. Moreover, some varieties of GFD do not necessarily lead to a healthier physiological state. Ercolini et al. [97], for example, showed that changing from an African-style GFD to an Italian-style GFD provoked significant changes in the salivary microbiota and metabolome of Saharawi (Western Sahara) celiac children and, more importantly, that these changes suggested metabolic dysfunction. 

Another issue with GFD is its availability and costs. While some regions in the industrialized world have the luxury of having access to a wide variety of foods and dietary ingredients, most regions of the world have limited access to different foods. This translates into wide differences in GFD, even within the same geographical region. Importantly, not all gluten-free products are certified (http://www.gfco.org/) and some supposedly gluten-free products are actually contaminated with gluten [98]. Moreover, the mere availability of dietary foods in one region does not imply that all people have access to it. In México, for example, differences in income may involve as much as 27-fold difference between the average incomes of the top and the bottom deciles, a difference that stark contrasts with the average ratio of 10 to 1 in the Organization for Economic Co-operation and Development (OECD 2014, https://www.worldeconomicsassociation.org/newsletterarticles/inequality-in-mexico/). Therefore, the purchasing power is likely to be involved in the maintenance of life-long GFD.

An interesting argument emerged from one anonymous reviewer during the review process. Indeed, other grains such as corn and rice are the primary grains consumed in many different countries. In the case of maize, which is often used as an alternative to elaborate GFD for CD patients, there are some maize prolamins (called zeins) containing amino acid sequences that resemble the wheat gluten peptides that may in fact be clinically relevant [99]. The case of rice is also interesting, especially because several countries in Asia have considerably reduced their consumption of rice [100] and increased the consumption of other grains. In addition, it has been recognized that CD epidemiology has changed, in particular in areas where previously CD was unrecognized or rare such as India, China and Latin America. Several hypotheses may explain this phenomenon such as a change in the pattern of food consumption to try to beat malnutrition that has led to a wheat–rice shift in poor countries, but dysbiosis and genetic predisposition may be also related [101]. Overall, we agree with the notion that the cost and availability of GFD may be of lesser clinical importance in some regions of the world. 

Aside the availability and costs, there is substantial variation in prescribing rates of gluten-free foods [102] and many GFD followers find it challenging to follow the GFD [103]. Interestingly, psychological state has been shown to predict adherence to a GFD in Australia and New Zealand [104]. Finally, a proportion of CD patients with chronic voluntary gluten ingestion do not show a relapse of clinical signs and villous atrophy despite chronic voluntary gluten ingestion [105], thus suggesting high inter-individual variability. This phenomenon is of great importance for the biomedical community, especially considering the variety of gluten-containing and GFD around the world (in other words, it is likely that not all diets have the same healing effect on regenerating enterocyte architecture and therefore health). 

## 6. Conclusions

The microbes that inhabit the human body are unique for each individual and vary widely over time due to multiple interrelated factors. The fact that the effect of GFD on health and the gut microbiota cannot be extrapolated to other populations is often neglected in the literature but can also apply to any dietary intervention in all other gastrointestinal maladies (e.g., IBD) associated with the gut microbiota. This paper shall not be taken as a discouragement to perform more studies on this topic; on the contrary, as mentioned above, this paper ultimately seeks to encourage the conception of more collaborative efforts to study local populations in an effort to reach useful conclusions that truly contribute to improve health in patients afflicted with GRD and other maladies. This paper also strives for more awareness among the medical community regarding potential negative effects of switching patients to a GFD without adequate dietetic and medical supervision.

## Figures and Tables

**Table 1:** Summary of studies that have analyzed the effect of gluten-free diet (GFD) on the gut microbiota and related parameters.

Patients Characteristics	Sample Collection and Analysis	Methods	Main Finding(s)	Reference (Year of Publication)
30 subjects (21–73 years old) with CD (*n* = 6), NCGS (*n* = 12) and controls (*n* = 12) from Veracruz, México	Samples were obtained at baseline and after 4 weeks on a GFD	16S rDNA sequencing using the Illumina MiSeq platform	*Pseudomonas* was higher in duodenum of CD patients after 4 weeks on GFD	[49] (Unpublished)
21 healthy adults (16–61 years old) from Groningen, The Netherlands	Nine samples were obtained from each participant at baseline, during and after 4 weeks on GFD	16S rDNA sequencing using 454 pyrosequencing	Veillonellaceae (class Clostridia, Firmicutes) was reduced on GFD; 21 predicted pathway activity scores showed significant association to the change in diet	[50] (2016)
53 young subjects (0.5–18 years old) with CD at presentation; 74 young subjects (1–18 years old) with CD on GFD for less than 1 year; 25 subjects (3–33 years old) with CD on GFD for more than 1 year from Norrköping, Sweden	One fecal sample was obtained once from each subject	Gas liquid chromatography for SCFA measurement	Fecal SCFA levels were higher in CD patients on GFD for < 1 year compared to those on GFD > 1 year	[51] (2013)
10 untreated CD patients, 11 treated CD patients and 11 healthy adults from Leon, Spain	Samples were obtained in normal gluten diet and in GFD	DGGE and gas-liquid chromatography of SCFAs	Microbial communities of treated CD clustered together with those of healthy adults	[52] (2012)
19 CD children (6–12 years old) on GFD for at least 2 years and 15 non-celiac children from Bari, Apulia, Italy	Duodenal biopsies and fecal samples were obtained once from each subject	DGGE and culture-based methods	2 years of GFD does not fully restore the microbiota and metabolome of CD children	[53] (2010)
24 untreated CD patients (2–12 years old) on a normal-gluten containing diet; 18 treated CD patients (1–12 years old) on GFD for at least 2 years; 20 healthy children (2–11 years old) without known gluten intolerance from Valencia, Spain	One fecal sample was obtained once from each subject	FISH, flow cytometry and immunoglobulin-coated bacterial analysis	CD patients have lower levels of IgA-coated bacteria thus providing new insights into the relationship between the gut microbiota and host immune defenses	[54] (2010)
20 children with CD (1.2–16.1years old) before and after at least 9 months on GFD, and 10 controls (7.8–20.8 years old) from Rome, Italy	Biopsies from the second part of the duodenum from CD children before and after at least 9 months on GFD; duodenal biopsies from the controls undergoing upper GI endoscopy for functional dyspepsia	TGGE	Number of bands was higher in active and inactive states compared to controls, implying higher biodiversity	[55] (2010)
10 healthy adults (23–40 years old) from Valencia, Spain	One fecal sample was obtained once from each subject at baseline and after 1 month on GFD	FISH and qPCR	Reduction of “beneficial” bacteria and the ability of fecal samples to stimulate the host’s immunity	[56] (2009)
34 CD patients at diagnosis and after 12 months on GFD, and 34 healthy controls from Fiorentino, Italy	Serum and urine samples were obtained once from each subject	Nuclear Magnetic Resonance (NMR) of urine and serum samples	After 12 months of GFD, all but one patient was classified as healthy	[57] (2009)
Group 1 (30 untreated CD patients on a normal gluten-containing diet, 56–61 months old); group 2 (18 treated CD patients with a GFD for at least 2 years, 64–58 months old); group 3 (30 control children without gluten intolerance, 45–49 months old) from Valencia, Spain	30 fecal and 25 duodenal biopsies from Group 1; 18 fecal and 8 biopsy samples from Group 2; 30 fecal and 8 biopsy samples from Group 3	qPCR for a small group of selected microbes	Duodenal and fecal microbiota is partially restored after long-term (>2 years) GFD	[58] (2009)
Seven symptom-free CD patients on GFD for at least 2 years; seven CD patients on gluten-containing diet; seven children with no known food intolerance (6–12 years old) from Bari, Apulia, Italy	Each child provided 3 fecal samples over an unknown period of time. The samples were mixed	DGGE and culture-based techniques; gas chromatography-mass spectrometry for VOCs	CD is associated with differences in fecal microbiota and biochemistry	[59] (2009)
20 CD patients (1.6–12 years old) and 10 symptom-free CD patients who had been on GFD for 1–2 years (2–8 years old) and 8 control children (2–7.8 years old) from Valencia, Spain	An unknown number of biopsy specimens was obtained once from each subject	FISH and flow cytometry for a few selected bacterial groups in duodenum	Ratio of *Lactobacillus*-*Bifidobacterium* to *Bacteroides*-*E. coli* was reduced in CD patients with either active or inactive disease compared to controls	[60] (2007)
36 children with CD at presentation, 47 patients on GFD for at least 3 months, and 42 healthy controls from Stockholm, Sweden	One fecal sample was obtained once for each subject	Gas-liquid chromatography of SCFAs in fecal samples	Difference between children on GFD and controls regarding acetic, i-butyric, i-valeric acid, and total SCFAs	[61] (2005)

CD, celiac disease; DGGE, Denaturing Gradient Gel Electrophoresis; TGGE, Temperature Gradient Gel Electrophoresis; FISH, Fluorescent in situ hybridization; SCFAs, short-chain fatty acids; VOCs, volatile organic acids; NCGS, non-celiac gluten sensitivity.
